# Annotations

**Published:** 1899-08-26

**Authors:** 


### ANNOTATIONS.

### Municipal Milk.

To St. Helens, Lancashire, seems due the credit of introducing into England the system of supplying sterilised milk in a form ready for infants' food, which has for some time been in operation in various towns abroad. How far the municipalisation of industry is to go is a question into which we will not enter, but if it is proper that our county councils and other public bodies should enter into the business of omnibus proprietors, tramway owners, gas makers, electric light furnishers, refreshment stall owners, and other trades too numerous to mention, we surely cannot say anything against this new product of civilisation—the municipal milkman—especially in view of the fact that so far the ordinary milkman of the independent type has failed to supply what is wanted, namely, milk fit for babes at a price fit for the pockets of the poor. For twopence the St. Helens Corporation supplies nine bottles of humanised milk, each containing one "feed," the idea being that the infant shall be fed every two hours during the day, and every four hours during the night. This milk is previously sterilised by exposure to steam at a temperature of 102 deg. C. for ten minutes. Of the efficacy of this measure in preventing the large mortality which occurs every autumn in some towns among bottle-fed babies there can be no doubt. It has been in operation for some time in New York, Paris, and various other places with the best results, and, of course, for years past the system of sterilising milk for infants' use in bottles, each containing one feed, has been in constant use both in hospital and in private practice. The difficulty has been to get it done. Our sympathy with suffering children makes us wish the new venture every success. It is hardly creditable, however, to the milk trade that it should have allowed the business to slip out of its hands. Why should we not all use sterilised milk instead of drinking stuff that has been slopped about by the milkman? It is all a matter of price. A slight reduction in the price of sterilised milk would produce a revolution in domestic management, and would at the same time save many lives and an untold amount in doctors' bills.

### Our Burial Laws.

We should have thought that the law as to burial was sufficiently simple, and that there was nowadays no chance of a person being buried without the death having been registered. Yet such seems to be the case, and, from the evidence given at a recent inquest, we might almost say such seems to be the system. The tale runs thus: A little child, the daughter of Italian parents, died under the care of an unqualified practitioner, who of course could not give a certificate. As, however, the child had previously been under the care of a medical man, he was applied to and he gave the required certificate, in which it somehow happened that the name inserted was not the correct name of the child. Perhaps this was an accident, but at any rate it led to a still further demonstration of the laxity which prevails in all that belongs to our burial customs. According to the evidence at the inquest this certificate was given to the undertaker along with the full name of the child. The undertaker appears, then, to have written to the superintendent of the cemetery arranging for the funeral to take place the next day, not noticing that the name he sent was different from the name on the certificate. Meanwhile the death was never registered, and of course no burial certificate was obtained, so that one might have thought that a double difficulty would have stood in the way of getting this child buried. Not at all. On the funeral arriving at the cemetery the medical certificate, a document which ought to have been sent to the registrar, and had nothing whatever to do with the cemetery people, was exhibited and the funeral was allowed to proceed; so that the burial took place not only without a proper certificate, but in spite of the fact that the medical certificate, which was improperly accepted in its place, bore a wrong name. Surely this is an almost incredible tangle of errors. But now comes the really serious part of the business. According to the evidence of the undertaker cases were often buried without the registrar's certificate, and it was possible for a person to be buried without a certificate at all. How this could happen was shown by the evidence given by the superintendent of the cemetery, who said that when a body had been buried on a doctor's certificate, and he had sent the registrar notice of it, still the certificate had never been forthcoming. On this the coroner remarked, "So that it might have been used to bury another person the next day in another part of London?" to which the witness answered, "Yes." And this is the way we bury our dead in the greatest city in the world! All our precautions in regard to registration are a farce if people can be buried without the authorisation of the registrar.

### Typhoid Fever in American Cities.

According to Dr. S. F. Crum,[1] the typhoid mortality rates of American cities are almost uniformly excessively high, although there has been a notable reduction in most of them during the last decade. In 24 American cities the average mortality from typhoid fever during the decade 1889-1898 was 41 per 100,000 living, which we may compare with that in London, from 1890 to 1898, which was but 13 per 100,000; and that in Berlin, which was only 7 per 100,000. If, however, we divide the decade into two, we find that a considerable reduction has taken place. Thus, during the first half of the period, the average typhoid mortality in these American cities was .52; while, during the second half, it only amounted to 32 per 100,000, a very considerable improvement. Still, even this shows a very serious prevalence of the disease, and one which is by no means creditable to municipal government in America. Dr. Crum states that where the mortality has been materially reduced, this has been due in most instances to improvements in the water supplies, and it seems to us that a careful consideration of the figures given affords the strongest possible evidence that the typhoid prevalence which is such a curse to American cities is not so much due to local causes as to defective protection of the sources of supply, and to defects in, or, indeed, to entire absence of filtration. Nothing else, except perhaps the uccurrence of milk outbreaks, would be at all likely to cause such extraordinary variations as are to be found in the returns for different years. Chicago is one year down to 28, and another is up to 173 per 100,000. Denver now has a mortality of 25, while nine years ago it stood at 269 per 100,000. Cleveland, again, oscillates from 176 to 21; Pittsburg, always very high, from 132 to 54; and so on. Such sudden variations point almost inevitably to fouling of the water supply. Endemic typhoid may vary, but not like this.

[1] New York Med. Rec., Aug. 12.

